# Safety of immune checkpoint inhibitor rechallenge after severe immune-related adverse events: a retrospective analysis

**DOI:** 10.3389/fonc.2024.1403658

**Published:** 2024-07-08

**Authors:** Céline Eldani, Marie Kostine, Maxime Faure, Estibaliz Lazaro, Claire Rigothier, Jean Baptiste Hiriart, Bérénice Teulières, Florian Poullenot, Magalie Haissaguerre, Maeva Zysman, Rémi Veillon, Charlotte Vergnenegre, Nahema Issa, Charlotte Domblides, Sorilla Mary-Prey, Marie Beylot-Barry, Anne Pham-Ledard, Caroline Dutriaux, Guilhem Sole, Fanny Duval, Emilie Gerard

**Affiliations:** ^1^ Department of Dermatology, Centre Hospitalier Universitaire (CHU) Bordeaux, Bordeaux, France; ^2^ Department of Rheumatology, CHU Bordeaux, Bordeaux, France; ^3^ Department of Cardiology, CHU Bordeaux, Bordeaux, France; ^4^ Department of Internal Medicine, Haut-Leveque, Bordeaux University Hospital, Bordeaux, France; ^5^ Department of Nephrology, Transplantation, and Dialysis, Bordeaux University Hospital Center, Bordeaux, France; ^6^ Hepatology Unit, Bordeaux University Hospital, Bordeaux, France; ^7^ Department of Gastroenterology and Hepatology, Centre Médico-chirurgical Magellan, Hôpital Haut-Lévêque, CHU de Bordeaux, Bordeaux, France; ^8^ Department of Endocrinology, University Hospital of Bordeaux, Bordeaux, France; ^9^ Pulmonary Department, Pôle Cardio-thoracique, CHU de Bordeaux, Bordeaux, France; ^10^ Intensive Care Unit and Infectious Disease, University Hospital Centre Bordeaux, Bordeaux, France; ^11^ Department of Medical Oncology, Bordeaux University Hospital, Bordeaux, France; ^12^ BRIC (BoRdeaux Institute of onCology), Institut National de la Santé et de la Recherche Médicale (INSERM), Univ. Bordeaux, Bordeaux, France; ^13^ AOC Referral Center for Neuromuscular Diseases, Neurology and Neuromuscular Diseases Department, FILNEMUS, University Hospitals of Bordeaux, Bordeaux, France

**Keywords:** immune checkpoint inhibitors (ICIs), immune related adverse events (irAEs), rechallenge, neurological immune-related adverse events, myocarditis

## Abstract

Immune checkpoint inhibitors (ICIs) present clinicians with the challenge of managing immune-related adverse events (irAEs), which can range from mild to severe due to immune system activation ^1^. While guidelines recommend discontinuing ICIs for grade 3 partial and all grade 4 irAEs, there is growing interest in rechallenging patients based on oncological outcomes, particularly for cardiovascular and neurological irAEs where data remains scarce ^1,2^. We retrospectively evaluated the safety of ICI rechallenge following grade 3-4 irAEs, specifically focusing on cardiovascular and neurological events, in patients discussed at our multidisciplinary immunotoxicity assessment board between 2019 and 2021. Fifteen patients were included, with a median time to severe irAE onset of 49 days. Among them, five patients experienced neurological adverse events (NAEs): aseptic meningitis (3), inflammatory polyradiculoneuropathy (1), and ophthalmoplegia (1), while one patient presented with myocarditis. Of the 15 patients retreated with ICIs after initial severe irAEs, 11 (73%) remained free of subsequent irAEs, two (13%) experienced recurrence of the initial irAE, and two (13%) developed new irAEs distinct from the initial event. The median time to event recurrence was 69 days, occurring no earlier than the initial severe irAE. In the subset analysis focusing on severe cardiovascular and neurological irAEs, rechallenge with ICIs was generally well tolerated. However, one patient treated with anti-PD1 experienced a relapse of grade 2 aseptic meningitis. Overall, our findings suggest that rechallenging with ICIs after severe irAEs, including those affecting the cardiovascular and neurological systems, may be safe, particularly after irAE regression and corticosteroid withdrawal.

## Introduction

Introduced 20 years ago, immune checkpoint inhibitors (ICIs) have revolutionized cancer treatment, leading to substantial improvements in cancer outcomes and overall survival rates. These agents, including anti-CTLA-4, anti-PD-1, and anti-PD-L1 therapies, have been widely adopted in clinical practice, either as monotherapy or in combination, for the treatment of advanced solid tumors and hematologic malignancies. However, their mechanism of action, which involves activating T-cells, can also lead to immune-related adverse events (irAEs) affecting various organs. Severe irAEs, classified as grades 3-4, are reported in approximately 8% of patients receiving anti-PD-1 monotherapy, 25% with anti-CTLA-4 monotherapy, and up to 50% with combination ICIs (1). Algorithms for managing these toxicities have been developed based on the affected organ system and the severity grade. Current guidelines recommend the use of high-dose systemic corticosteroids and permanent discontinuation of ICIs for grades 3-4 irAEs (1, 2). For patients who discontinue ICI due to severe toxicity, the question of rechallenge becomes pertinent. This decision is complex and relies on the specialist’s assessment of the risk-reward ratio and the availability of alternative treatment options, often deliberated in a multidisciplinary setting. Despite the growing interest in ICI rechallenge, there is a paucity of data, particularly regarding severe neurological and cardiac toxicities (3, 4). Therefore, it is imperative to elucidate the clinical characteristics and outcomes of patients who undergo ICI rechallenge after experiencing severe irAEs. Such insights are crucial for informing clinical decision-making and optimizing the management of irAEs in patients receiving ICIs.

## Methods

We conducted a retrospective monocentric study to investigate the rechallenge of ICIs following the occurrence of an initial grade 3 or 4 irAE. Patients were identified from the database of the multidisciplinary immunotoxicity assessment board (ImmunoTox) at Bordeaux University Hospital between December 2019 and December 2021. This board comprises a nationwide network of oncologists, immunologists, and organ specialists with expertise in managing irAEs.

Inclusion criteria encompassed patients treated with either single ICI (anti-PD-1, anti-PD-L1) or combination ICIs (anti-PD-1 + anti-CTLA-4), who experienced an initial severe toxicity graded as 3 or 4 according to the National Cancer Institute Common Terminology Criteria for Adverse Events. Board agreement for ICI rechallenge was mandatory for inclusion. Exclusion criteria were limited to cases with missing follow-up data or those where rechallenge did not occur despite board agreement.

We collected comprehensive data including patients’ socio-medical characteristics, type of initial ICI administered, characteristics and grading of the initial irAE, management strategies employed, and outcomes of the irAEs. Additionally, details regarding the second ICI used for rechallenge, disease status (progression, partial response, or stability) prior to rechallenge, and any concurrent immunosuppressive therapy (such as corticosteroids exceeding 20 mg/day or other immunosuppressive agents) were recorded.

This study design allowed for a thorough examination of the clinical parameters surrounding ICI rechallenge after severe irAEs, providing valuable insights into patient management and outcomes in this challenging clinical scenario.

## Results

Among the 112 patients referred to our board during the study period, 28 patients were approved for ICI rechallenge following the occurrence of severe irAEs. However, 13 patients were excluded due to follow-up outside Bordeaux University Hospital (n=8), chemotherapy combined with immunotherapy (n=3), or failure to undergo rechallenge despite board agreement (n=2). Ultimately, our analysis included 15 patients who underwent ICI rechallenge after experiencing grade 3 or higher irAEs.

Patient characteristics are summarized in **Table 1**. The initial ICI regimen comprised anti-PD-1/anti-PD-L1 for 9 patients and combination ICIs for 6 patients. The median time from ICI initiation to initial toxicity onset was 49 days (interquartile range, 17-246 days). Detailed characteristics, management, and outcomes of the initial irAEs are presented in **Table 2**.

**Table 1:** Demographic and oncological characteristics of patients with rechallenge ICI after severe irAE.

		n=16
Age median (range)		61 (33-88)
Sex (%)	Male	8 (53)
	Female	7 (47)
Primary tumor site (%)	Cutaneous melanoma	13 (86.8)
	Mucosal melanoma	1 (6.6)
	Lung	1 (6.6)
Autoimmune disease (%)	Ulcerative colitis	1 (6.6)
	Psoriatic arthritis	1 (6.6)
	None	13 (86.8)
First ICI (%)	Pembrolizumab	4 (26.7)
	Nivolumab	4 (26.7)
	Atezolizumab	1 (6.6)
	Ipilimumab+Nivolumab	6 (40)
Time to irAE in days, median (range)		49 (17-246)
Grade of initial irAE (%)	Grade 3	15 (100)
	Grade 4	0 (0)
Systemic corticosteroids use (%)	Yes Duration in months, median (range)	13 (87) 5,5 (2-8)
	No	2 (13)
Other treatment for irAE	Intravenous immunoglobulins	2
	Methotrexate	2
	Infliximab	1
	Hydroxycloroquine	1
	Antibiotics	2
Hospitalization (%)	Yes	14 (93.4)
	Duration in days, median (range)	13 (4-20)
	No	1 (6.6)

ICI, immune checkpoint inhibitors; irAE, immune related adverse events.

**Table 2:** Recurrence of irAEs after ICI rechallenge.

Side effect	n=16	initial ICI	Additional tests	Treatment	Evolution	Grade	Rechallenge	Relapse after rechallenge
**Neurologic** Aseptic meningitis	3	anti PD-1 monotherapy	LP: lymphocytic meningitis	CTC 0.5 mg/kg/day p.o.	Healing	0	anti PD-1 + anti CTLA-4 + CTC 0.75 mg/kg/day	Same irAE: aseptic meningitis grade 2 + New irAE: hypoxemic pneumonia grade 2
		anti PD-1 + anti CTLA-4	LP: lymphocytic meningitis	CTC 3 days course of 250 mg IV then relay 1 mg/kg/day p.o.	Improvement	1	anti-PD-1 monotherapy + CTC 20 mg/day for 15 days then gradual decrease	No
		anti PD-1 + anti CTLA-4	LP: lymphocytic meningitis	CTC 3 days course of 250 mg IV then relay 0.5 mg/kg/day p.o.	Healing	0	anti-PD-1 monotherapy without CTC	No
Inflammatory polyradiculoneuropathy	1	anti PD-1 monotherapy	EMG: demyelinating injury, antiganglioside and onconeural antibodies negatives	IVIG 5 days course of 150 g X2	Improvement	1	With another anti-PD-1 + CTC 10 mg/day p.o.	No
Ophthalmoplegia/impaired neuromuscular junction	1	anti PD-1 monotherapy	LP: lymphocytic pleocytosis	CTC 5 days course of 500 mg IV And IVIG 0,4g/kg/day	Improvement	1	another anti-PD-1 + CTC 10 mg/day p.o. + CPK monitoring	No
**Cardiac** Myocarditis	1	anti PD-1 monotherapy	Troponin 75 ng/L, cardiac ultrasound: pericardial effusion,	CTC 3 days course of 1000 mg IV then relay 2 mg/kg/day p.o.	Healing	0	With another anti-PD-1 + CTC 30 mg/day	No
**Respiratory** Hypoxemic pneumonia	2	anti PD-1 monotherapy	CT scan: interstitial lung disease, BALF: lymphocytosis,	CTC 1 mg/kg/day p.o. + piperacillin-tazobactam 4 g X4/day	Healing	0	With another anti-PD-1 + CTC 1 mg/kg/day (for brain metastases)	No
		anti PD-1 + anti CTLA-4	CT scan: interstitial lung disease BALF: lympocytosis	CTC 0.75 mg/kg/day p.o. + piperacillin-tazobactam 4 g X4/day	Improvement	1	anti-PD-1 monotherapy + CTC 30 mg/day	Same irAE: hypoxemic pneumonia grade 2 + New irAE: lichenoid dermatitis grade 2
**Digestive** Colitis	2	anti PD-1 + anti CTLA-4	FOGD-colonoscopy normal, aspecific colon biopsies	CTC 0,5 mg/kg/day p.o.	Healing	0	anti-PD-1 monotherapy +CTC 0,5 mg/day p.o.	New irAE: lichenoid dermatitis grade 1
		anti PD-1 monotherapy	Endoscopy: severe rectosigmoiditis with deep ulcerations	CTC 1 mg/kg/day p.o. + infliximab 5 mg/kg at week W0, W2 and W6 then every 8 weeks	Improvement	1	With a same anti-PD-1 + CTC 40 mg/day + infliximab	No
**Skin** Bullous pemphigoid	1	anti PD-1 monotherapy	Skin biopsy: subcutaneous detachment with many eosinophils, positive direct immunofluorescence, positive anti-basal membrane antibodies	Topical clobetasol + methotrexate 7.5 mg/week p.o.	Healing	0	With a same anti-PD-1 + Topical clobetasol + methotrexate 7,5 mg/week p.o.	No
Lichenoid dermatitis	1	anti PD-1 monotherapy	Skin biopsy: mixed subepidermal blister, lichenoid lesions and few necrotic keratinocytes, negative direct immunofluorescence	CTC 1 mg/kg/day p.o. + topical clobetasol	Healing	0	With another anti-PD-1 + CTC 1 mg/kg/day p.o.	No
**Hepatic** Hepatitis	1	anti PD-1 + anti CTLA-4	Transaminitis 17X ULN without elevated bilirubin, liver biopsy: consistent with acute immune-induced hepatitis	CTC 0.5 mg/kg/day p.o.	Healing	0	anti-PD-1 monotherapy without CTC	New irAE: thrombopenia grade 1
**Rheumatology** Relapse of rheumatism	1	anti PD-1 + anti CTLA-4	Ultrasound: arthritis	CTC 20 mg/day + methotrexate 20 mg/week p.o. + hydroxychloroquine 400 mg/day + hydrocortancyl infiltration in joint	Improvement	1	anti-PD-1 monotherapy + CTC 20 mg/day	No
**Hematologic** Macrophage activation syndrome	1	anti PD-1 monotherapy	hyperferritinemia, elevated triglycerides and LDH, hepatic cytolysis, moderate cytopenia, normal myelogram, liver biopsy: signs of hemophagocytosis	CTC 1 mg/kg/day p.o.	Healing	0	With a same anti-PD-1 Without CTC	No
**Endocrine** Diabetes with ketoacidosis	1	anti PD-1 + anti CTLA-4	Blood glucose 7g/L, pH 7.08, low bicarbonate levels	Insulin IV	Improvement	1	anti-PD-1 monotherapy	No

With n=1 Diabetes with ketoacidosis + aseptic meningitis.

ICI, immune checkpoint inhibitor; LP, lumbar puncture; CTC, corticosteroids; MRI, magnetic resonance imaging; EMG, electromyography; IVIg, intravenous immunoglobulin; CK, creatine kinase; AChR, acetylcholine receptor; BALF, bronchoalverolar lavage fluid.

Among the 15 patients, 5 (33%) developed neurological adverse events (NAEs), including aseptic meningitis (n=3), inflammatory polyradiculoneuropathy (n=1), and neurological ophthalmoplegia (n=1). Prompt treatment with systemic corticosteroids and/or intravenous immunoglobulins (IVIg) resulted in complete recovery for 2 patients. However, sequelae persisted in cases of inflammatory polyradiculoneuropathy and ophthalmoplegia.

Additionally, one patient presented with immune-induced myocarditis, which was promptly managed with high-dose intravenous corticosteroids, leading to clinical and biological improvement. Other initial severe irAEs resulting in treatment interruption included pneumonitis, colitis, rash, hepatitis, rheumatoid arthritis, macrophage activation syndrome, and endocrine disorders.

ICI treatment was discontinued for all 15 patients due to severe irAEs. Most patients (87%) received systemic corticosteroid therapy, with a median hospitalization duration of 13 days (IQR: 4-20 days). In our study, 13 out of 16 patients treated with systemic corticosteroids for immune-related adverse events (irAEs) responded positively, showing healing or improvement. The corticosteroid doses varied, typically starting with high initial doses (e.g., 250-1000 mg IV) followed by maintenance doses (e.g., 0.5-2 mg/kg/day p.o.), with treatment duration adjusted based on clinical response. Two patients did not respond to corticosteroid therapy, either developing new irAEs or not achieving improvement. Three patients were managed without corticosteroids, with one showing improvement with alternative treatment.

Rechallenge strategies varied, with the majority (66%) initiated due to cancer progression. At the time of rechallenge, 7 patients were under immunosuppressive therapy.

Overall, only 2 patients (13%) experienced recurrence of the same irAE, with an additional 2 patients (13%) developing new irAEs. The median duration of irAE occurrence after rechallenge was consistent with the initial event (69 days; IQR: 54-168 days). Median follow-up after rechallenge was 8 months (range: 6-68 months), with varying responses observed, including complete response, partial response, stability, and cancer-related deaths.

## Discussion

In our study, 11 out of 15 patients (73%) who experienced initial severe irAEs did not encounter a recurrence of irAE after ICI rechallenge. Although the initial cohort consisted of 112 patients, the final sample size was reduced to 15 due to stringent inclusion criteria, focusing on patients with comprehensive data on irAEs and their treatment outcomes. This selective approach, while limiting the sample size, ensures a high-quality dataset and enhances the reliability of our findings.

The safety of rechallenge following grade 2 or higher irAEs, particularly involving neurological or cardiac toxicities, remains inadequately explored in the literature (3–5). Pollack et al. demonstrated that among 80 patients who underwent anti-PD-1 rechallenge following irAEs (69% grade 3 or 4) during combination ICIs for metastatic melanoma, 18% experienced recurrence of the initial irAE, 21% developed a different irAE, and 61% did not encounter irAE recurrence (6). Notably, this study did not include initial neurological, cardiac, or muscular irAEs. Similarly, Simonaggio et al. reported on 40 patients undergoing anti-PD-1 rechallenge after irAEs (54% grade 3 or 4) during initial anti-PD-1 therapy for various cancers, with 42.5% experiencing recurrence of the initial irAE, 12.5% developing a different irAE, and 45% not encountering irAE recurrence. Notably, retreatment was avoided for life-threatening toxicities such as cardiac irAEs, neurological adverse events (NAE), and severe myositis (3). Neurological toxicities associated with anti-PD-1 therapy alone or in combination with anti-CTLA-4 agents have been reported with varying incidence rates, ranging from 0.5% to 6.1% (7).

Rechallenge after neurological toxicities remains rare, often considered as a last-line treatment following cancer progression and failed alternative therapies. Persistent neurological symptoms, often graded as 1 or 2, are frequently observed despite high-dose corticosteroids and IVIg therapy, resembling ICI sequelae (8, 9). Park et al. reported on ICI rechallenge in patients with initial myocarditis and NAE, with no flare or toxicity observed in 45.2%, recurrence of the rare irAE in 22.6%, and a different irAE in 37.1% (10). Similarly, Weill et al. described ICI rechallenge in patients with immune-related myositis, with no myositis relapse observed and only one patient experiencing a different irAE (11).

Pharmacovigilance data indicate a low incidence of myocarditis with immune checkpoint inhibitors (ICIs), ranging from 0.27% to 0.9%, with higher rates in combination therapy and anti-PD-1/anti-CTLA-4 therapy. Risk factors such as underlying autoimmune diseases and cardiovascular conditions may increase susceptibility, though differences in incidence between ICI classes are not significant, suggesting a similar toxicity profile among agents (12). The ASCO guidelines advise against ICI therapy rechallenge following grade 1 myocarditis due to its severity, emphasizing individualized decision-making with cardiologists and oncologists, supported by limited literature consisting of a few case reports and series, warranting rigorous monitoring if rechallenge is contemplated (1). The study of Frascaro and al. aimed to review the literature on rechallenging immunotherapy after myocarditis caused by ICI (13). Nine case reports involving 16 patients were identified, showing varied outcomes ranging from successful rechallenge to recurrent myocarditis or cancer progression. In four cases, rechallenging with ICI after myocarditis led to ineffective outcomes, with cardiac-related issues such as myocarditis recurrence or worsening symptoms. Notably, patients initially diagnosed with severe myocarditis did not encounter these challenges, suggesting additional factors influence relapse risk. Additionally, in two cases, patients experienced fatal cancer progression despite rechallenge, emphasizing the critical need for thorough risk-benefit assessment by oncologists and further clinical studies on this complex clinical scenario. These findings underscore the importance of carefully assessing the risk-benefit ratio before considering rechallenge and highlight the need for further research in this area.

In our study, rechallenge following severe neurological toxicities resulted in one recurrence out of five cases, while rechallenge after myocarditis did not lead to recurrence. Notably, recurrence of NAE (aseptic meningitis) prompted rechallenge with combination ICIs and high-dose corticosteroids. Conversely, cases without recurrence of NAE did not involve high-dose corticosteroids during rechallenge, often opting for ICI monotherapy. Data indicated a significantly higher risk of irAE recurrence in patients receiving corticosteroids exceeding 20 mg/day at rechallenge, possibly due to masking of unresolved toxicity. Therefore, complete regression or at least grade 1 toxicity before rechallenge is crucial. Nonetheless, rechallenge after neurological or cardiac toxicity remains a viable option under vigilant monitoring.

## Data availability statement

The raw data supporting the conclusions of this article will be made available by the authors, without undue reservation.

## Ethics statement

Ethical approval was not required for the study involving humans in accordance with the local legislation and institutional requirements. Written informed consent to participate in this study was not required from the participants or the participants’ legal guardians/next of kin in accordance with the national legislation and the institutional requirements. Written informed consent was obtained from the individual(s) for the publication of any potentially identifiable images or data included in this article.

## Author contributions

CE: Writing – original draft. MK: Writing – review & editing. MF: Writing – review & editing. EL: Writing – review & editing. CR: Writing – review & editing. JH: Writing – review & editing. BT: Writing – review & editing. FP: Writing – review & editing. MH: Writing – review & editing. MZ: Writing – review & editing. RV: Writing – review & editing. CV: Writing – review & editing. NI: Writing – review & editing. CDo: Writing – review & editing. SM-P: Writing – review & editing. MB-B: Writing – review & editing. AP-L: Writing – review & editing. CDu: Writing – review & editing. GS: Writing – review & editing. FD: Writing – review & editing. EG: Writing – review & editing.
